# National Evaluation of the Management of Melanoma Patients with Multiple Positive Sentinel Lymph Nodes

**DOI:** 10.1245/s10434-025-18466-4

**Published:** 2025-11-01

**Authors:** Tyler P. Robinson, Kristen N. Kaiser, Brian Ruedinger, John Hyngstrom, Theodore F. Logan, William A. Wooden, Ryan J. Ellis, Karl Y. Bilimoria

**Affiliations:** 1 Surgical Outcomes and Quality Improvement Center (SOQIC), Department of Surgery, Indiana University School of Medicine, Indianapolis, IN USA; 2 Division of Surgical Oncology, Department of Surgery, Rush University Medical College, Chicago, IL USA; 3 Indiana University School of Medicine and IU Simon Cancer Center, Indianapolis, IN USA; 4 Division of Plastic and Reconstructive Surgery, Department of Surgery, Indiana University, Indianapolis, IN USA

## Abstract

**Introduction:**

Clinical trials for melanoma patients demonstrated safety of observation in lieu of completion lymph node dissection (CLND) following positive sentinel lymph node (SLN) biopsy. Patients with two or more positive SLNs were infrequently included in the trials, leaving uncertainty about their management. We aimed to (1) assess national trends of CLND use in patients with two or more positive SLNs; (2) examine factors associated with CLND use; and (3) examine overall survival outcomes.

**Methods:**

Patients with stage I–III melanoma who underwent SLN biopsy between 2012 and 2021 were identified from the National Cancer Database. Factors associated with CLND were assessed by hierarchical logistic regression. Overall survival was estimated using Cox proportional hazards models.

**Results:**

Among 151,442 patients (median age 61 years; 41.3% female) who underwent SLN biopsy, 5440 (3.6%) had two or more positive SLNs. CLND in patients with two or more positive SLNs decreased from 73% in 2012 to 14% in 2021, while immunotherapy utilization increased from 38% in 2012 to 76% in 2021. Patients with two or more positive SLNs were more likely to undergo CLND if they had melanoma in the head/neck region (odds ratio [OR] 2.02, 95% confidence interval [CI] 1.39–2.95) or ulcerated lesions (OR 1.42, 95% CI 1.11–1.83). There was no difference in 3-year overall survival (70.5% for two or more positive SLNs with observation vs. 70.8% for two or more positive SLNs with CLND; hazard ratio 1.03, 95% CI 0.77–1.37) for observation vs. CLND.

**Conclusion:**

Utilization of CLND declined for melanoma patients with two or more positive SLNs following major clinical trials, with no difference in overall survival for observation versus CLND. Evolving treatment recommendations have been rapidly incorporated into practice in the United States.

**Supplementary Information:**

The online version contains supplementary material available at 10.1245/s10434-025-18466-4.

Historically, patients with positive sentinel lymph node (SLN) biopsies for melanoma underwent completion lymph node dissection (CLND) as the mainstay of management; however, CLND has been shown to have significant morbidity,^1^ and many patients who underwent CLND are ultimately found to have no further disease in non-sentinel nodes.^2,3^ This has led clinicians and researchers to question the clinical benefit of performing CLND following identification of positive SLNs and may contribute to the wide variability in melanoma practice patterns.^4^

The Multicenter Selective Lymphadenectomy II (MSLT-II) trial published in 2017, and the Dermatologic Cooperative Oncology Group (DeCOG) trial published in 2016 examined outcomes in positive SLN patients randomized to CLND versus observation.^5,6^ In both MSLT-II and DeCOG, complications were higher in patients who underwent CLND when compared with observation.^5,6^ While the rate of disease-free survival was higher in CLND compared with observation in the MSLT-II trial, the rate of 3-year melanoma-specific survival was similar between CLND versus observation in MSLT-II.^5^ The DeCOG trial found similar distant metastasis-free survival, recurrence-free survival, and overall survival when comparing CLND with observation, which persisted on the final analysis with extended follow-up.^6,7^ Given comparable outcomes, nodal observation has become an acceptable clinical approach^8^ in patients with positive SLN disease.^5,6^ Furthermore, immunotherapy has become a major component of treatment for melanoma. Clinical trials examining the efficacy of immunotherapy in melanoma patients were conducted around the same time as MSLT-II and DeCOG, which has also impacted management for patients with melanoma.^9–15^

However, a majority of patients in MSLT-II (70%) and DeCOG (90%) had only one positive SLN; thus, it is unknown how these trials have been interpreted regarding the use of CLND in patients with two or more positive SLNs.^16^ Therefore, the objectives of this study were to (1) assess national patterns of CLND use in patients with two or more positive SLNs; (2) examine factors associated with CLND use; and (3) examine overall survival in patients with two or more positive SLNs who undergo observation versus CLND. Additionally, this study examined uptake of immunotherapy use in this population given that these two changes in practice, use of CLND and immunotherapy, were occurring concurrently.

## Methods

### Patient Selection

The National Cancer Database (NCDB), a joint effort between the American College of Surgeons Commission on Cancer (CoC) and the American Cancer Society, was the data source for this analysis.^17^ Data collected by more than 1300 CoC-accredited facilities were included within the NCDB 2021 melanoma Participant Use File (PUF), the most recent year the NCDB has released data on melanoma. These data are de-identified, Health Insurance Portability and Accountability Act (HIPPA)-compliant, and are made available to CoC-affiliated institutions. The Institutional Review Board (IRB) office deemed that this did not require review as it is non-human subjects research. Within the NCDB, we identified patients with melanoma who had been treated between 1 January 2012 and 31 December 2021 with International Classification of Diseases for Oncology, Third Edition (ICD-0-3), site codes 440–449, and ICD-0-3 histology codes 8720–8723, 8730, 8740–8745, 8770–8772, and 8780. We excluded patients who had carcinoma in situ, metastatic disease, unknown/not applicable staging, and clinically positive nodes.

### Surgery and Nodal Evaluation

We used definitions of surgery and nodal evaluation that were consistent with the CoC’s Facility Oncology Registry Data Standards (FORDS) and Standards for Oncology Registry Entry (STORE) site-specific procedure coding.^18^ Registrars from CoC hospitals abstract cases using standardized criteria.^18^ Regional lymph node surgery was coded as SLN biopsy, CLND, or regional lymph node dissection without SLNB biopsy using the variable ‘Scope of Regional Lymph Node Surgery 2012’. This variable was created in 2012 through efforts by the National Cancer Institute’s Surveillance Epidemiology and End Results (SEER), the CoC, and the Center for Disease Control and Prevention’s National Program of Cancer Registries (CDC/NPCR) to improve the accuracy of SLN biopsy and CLND reporting.^19^ To identify positive SLNs, we utilized the variable ‘Sentinel Lymph Nodes Positive’, introduced in 2018 to provide increased granularity while differentiating positive regional lymph nodes from SLNs. We used the variable ‘Regional Lymph Nodes Positive’ when values for ‘Sentinel Lymph Nodes Positive’ were not available, which has been done in a similar analysis.^20^ Stage of melanoma was determined using the variable ‘NCDB Analytic Stage Group’, which is assigned the value of the 8th American Joint Committee on Cancer (AJCC) pathologic stage, whereas the 8th AJCC clinical stage is used if the pathologic stage is not reported.

### Immunotherapy

To identify trends in immunotherapy utilization, patients who underwent, or were intended to receive, immunotherapy as first-course treatment were included. Immunotherapy is defined as biological or chemical agents that alter the immune system or change the host’s response to tumor cells.^18^

### Outcomes

The primary outcome of this study was CLND in patients with two or more positive SLNs. Although trends from 2012 to 2021 were examined, we were specifically interested in CLND practice patterns following the MSLT-II and DeCOG clinical trials; therefore, we selected the years 1 January 2018 to 31 December 2021 for the subsequent analyses. This time interval may allow for dissemination of MSLT-II and DeCOG clinical trial results and subsequent adoption into national practice patterns. Given the concomitant uptake of immunotherapy with changes in CLND use, immunotherapy practice patterns and overall survival in patients with two or more positive SLNs were examined.

### Statistical Analysis

Continuous data were analyzed using the Mann–Whitney test, and categorical data were analyzed using the Chi-square test. Hierarchical logistic regression modeling with clustering at the facility level to account for non-independence of patients within hospitals was performed to identify patient, tumor, and hospital factors associated with the use of CLND. MSLT-II/DeCOG trial results regarding nodal observation became widely available in 2017; therefore, the years 2018–2021 were used to examine subsequent practice patterns. Model variables included demographic characteristics such as age, race/ethnicity, insurance status, and Charlson–Deyo comorbidity score. Zip code-level variables for median income and high-school education were included, as well as facility melanoma case volume, facility type (academic or non-academic), patient distance traveled to the reporting facility (50-mile cut-off similar to previous work^20–24^), country region of the reporting facility, and population type where the reporting facility was located (metro, urban, rural). Cancer features such as Breslow depth, ulceration status, primary tumor location, lymphovascular invasion, and immunotherapy use were also included. Odds ratios (ORs) and confidence intervals (CIs) were estimated. Kaplan–Meier survival curves were created and compared using log-rank analysis. Cox hazard modeling utilized the years 2018–2020 due to survival data for 2021 not being reported yet, and adjustments were made for the same variables as those in the multivariable logistic regression model. Tests were conducted with two-sided results and α = 0.05 was considered statistically significant. All analyses were carried out using STATA 18.0 (StataCorp LP, College Station TX, USA)

## Results

**Fig. 1:**
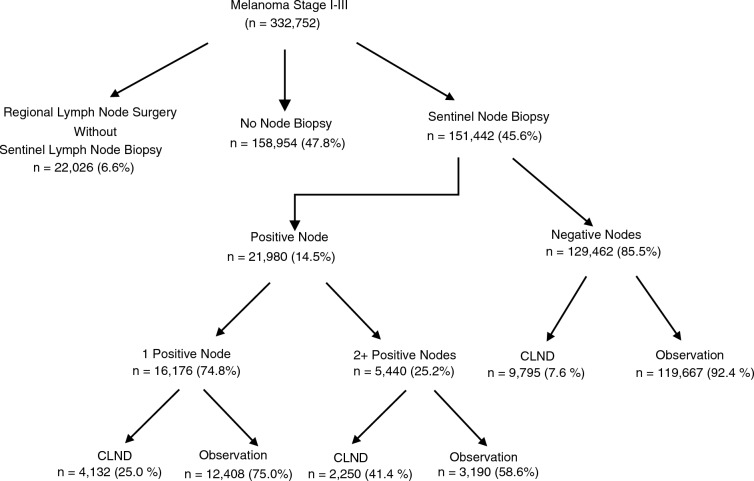
Management of patients with melanoma who underwent SLNB (2012–2021)

Among the 332,752 patients with stage I–III melanoma and clinically node-negative disease between 2012 and 2021, 151,442 (45.6%) underwent SLN biopsy with a median of two nodes removed, 52,321 (35.1%) had one SLN excised, and 96,780 (63.3%) had two or more SLNs excised. Of the patients who underwent SLN biopsy, 21,980 (14.5%) had a positive lymph node, including 16,176 (74.8%) with one positive SLN and 5440 (25.2%) with two or more positive SLNs (Fig. 1).

Patients who had two or more positive SLNs were more frequently male (62.2% vs. 58.4% for one positive SLN), under age 50 years (26.0% vs. 24.4% for one positive SLN), or identified as Hispanic race (3.2% vs. 2.5% for one positive SLN). Additionally, patients who had two or more positive SLNs had higher rates of private insurance (51.1% vs. 50.0% for one positive SLN), were treated at hospitals with the highest hospital volumes (25.5% vs. 22.4% for one positive SLN), were treated at academic centers (43.7% vs. 42.6% for one positive SLN), or traveled ≥50 miles (34.4% vs. 32.4% for one positive SLN) (Table 1). Patients with two or more positive SLNs more frequently had tumors with >4 mm Breslow thickness (30.9% vs. 22.4% for one positive SLN), ulceration (52.0% vs. 44.5% for one positive SLN), and lymphovascular invasion (20.8% vs. 12.9% for one positive SLN) (Table 2). Other patient demographics, hospital characteristics, and tumor characteristics are found in Tables 1 and 2.

**Table 1:** Descriptive characteristics of all patients who underwent SLNB, stratified by patients with one positive SLN versus those who had two or more positive SLNs

Characteristic	All patients receiving SLNBx^a^ [*n* = 151,442]^b^	Patients with one positive SLN [*n* = 16,176]	Patients with two or more positive SLNs [*n* = 5440]
*Sex*			
Male	88,884 (41.3)	9454 (58.4)	3384 (62.2)
Female	62,558 (58.7)	6722 (41.6)	2065 (37.8)
*Age, years*			
Median (IQR)	63 (52–73)	62 (50–72)	61 (49–71)
<50	31,060 (20.5)	3945 (24.4)	1414 (26.0)
50–59	29,743 (19.6)	3278 (20.2)	1162 (21.4)
60–69	40,169 (26.5)	3926 (24.3)	1362 (25.0)
70–79	33,690 (22.3)	3253 (20.1)	963 (17.7)
80+	16,780 (11.1)	1774 (11.0)	539 (9.9)
*Race*			
Non-Hispanic White	144,121 (95.6)	15,191 (94.2)	5038 (92.8)
Non-Hispanic Black	749 (0.5)	138 (0.9)	60 (1.1)
Hispanic	2344 (1.6)	397 (2.5)	180 (3.2)
Asian	511 (0.3)	93 (0.6)	37 (0.7)
Other	3717 (2.1)	297 (1.8)	125 (2.1)
*Insurance status*			
Medicare	65,048 (43.4)	6601 (41.2)	2080 (38.6)
Medicaid	4958 (3.3)	796 (5.0)	317 (5.9)
Private	74,954 (50.0)	8021 (50.0)	2752 (51.1)
Uninsured	2673 (1.8)	380 (2.4)	149 (2.8)
Other government	2266 (1.5)	235 (1.5)	89 (1.7)
*Charlson–Deyo score*			
0	124,611 (82.3)	12,966 (80.1)	4384 (80.6)
1	18,983 (12.5)	2196 (13.6)	738 (13.6)
2+	7848 (5.2)	1014 (6.3)	318 (5.9)
*Year*			
2012–2017	82,375 (54.4)	7131 (44.1)	2711 (49.8)
2018–2021	69,067 (45.6)	9045 (55.9)	2729 (50.2)
*Median household income*			
Lowest	13,215 (8.7)	1492 (9.2)	520 (9.6)
Low	24,519 (16.2)	2801 (17.3)	940 (17.3)
High	30,857 (20.4)	3340 (20.6)	1095 (20.1)
Highest	56,918 (37.6)	5587 (34.5)	1788 (32.9)
Unknown	25,933 (17.1)	2956 (18.3)	1097 (20.2)
*No high-school degree*			
Lowest	37,569 (29.9)	3657 (27.6)	1128 (25.9)
Low	41,068 (32.7)	4303 (32.5)	1410 (32.4)
High	31,385 (25.0)	3438 (25.9)	1183 (27.2)
Highest	15,638 (12.4)	1855 (14.0)	631 (14.5)
*Hospital volume of cases (mean)*			
Lowest (<13 cases/year)	33,105 (21.9)	3502 (21.6)	1106 (20.3)
Low (13–26 cases/year)	27,602 (18.2)	2723 (16.8)	891 (16.4)
Moderate (27–54 cases/year)	30,480 (20.1)	3117 (19.3)	957 (17.6)
High (55–109 cases/year)	30,055 (19.9)	3208 (19.8)	1100 (20.2)
Highest (≥110 cases/year)	30,200 (19.9)	3626 (22.4)	1386 (25.5)
*Facility type*			
Academic	63,535 (42.0)	6888 (42.6)	2377 (43.7)
Non-academic	87,907 (58.0)	9288 (57.4)	3063 (56.3)
*Distance traveled, miles*			
≤50	106,148 (70.1)	10,937 (67.6)	3566 (65.6)
>50	45,294 (29.9)	5239 (32.4)	1874 (34.4)
*Region*			
Northeast	27,110 (17.9)	2747 (17.0)	849 (15.6)
Midwest	35,436 (23.4)	4199 (26.0)	1401 (25.7)
South	48,284 (31.9)	4726 (29.2)	1593 (29.3)
West	26,435 (17.4)	2628 (16.2)	903 (16.6)
Unknown	14,177 (9.4)	1876 (11.6)	694 (12.8)
*Population density*			
Metro	123,946 (84.3)	12,985 (82.9)	4363 (83.0)
Urban	20,628 (14.0)	2395 (15.3)	813 (15.5)
Rural	2412 (1.6)	275 (1.8)	83 (1.6)

Data are expressed as *n* (%) unless otherwise specified

*SLN* sentinel lymph node, *SLNB* sentinel lymph node biopsy, *IQR* interquartile range

^a^Missing data: race/ethnicity (609), education (25,774), insurance (1543), population density (4456)

^b^Not all values add to 100% due to missing data

**Table 2:** Melanoma characteristics for all patients who underwent SLNB, stratified by patients with one positive SLN versus those with two or more positive SLNs

Characteristic	All patients receiving SLNBx^a^ [*n* = 151,442]^b^	Patients with one positive SLN [*n* = 16,176]	Patients with two or more positive SLNs [*n* = 5070]	*p*-Value
*Breslow thickness, mm*				<0.001
≤1.0	41,245 (28.2)	1631 (10.3)	313 (5.9)	
1.01–2.00	55,313 (37.4)	5084 (32.1)	1309 (24.7)	
2.00–4.00	33,497 (22.9)	5573 (35.2)	2044 (38.5)	
>4.00	16,113 (11.0)	3550 (22.4)	1640 (30.9)	
*Ulceration status*				<0.001
No ulceration	95,672 (65.7)	8764 (55.5)	2537 (48.0)	
Ulceration	49,951 (34.3)	7019 (44.5)	2751 (52.0)	
*Location of tumor*				<0.001
Head/neck	30,203 (20.0)	2348 (14.5)	889 (16.3)	
Trunk	47,179 (31.1)	5860 (36.2)	2058 (37.8)	
Upper extremity	43,187 (28.5)	3865 (23.9)	964 (17.7)	
Lower extremity	30,164 (19.9)	4010 (24.8)	1494 (27.5)	
Other/NOS	709 (0.5)	93 (0.6)	35 (0.6)	
*Immunotherapy*				<0.001
Given or recommended	14,649 (9.7)	8149 (50.6)	3124 (57.7)	
Not given	136,561 (90.3)	7961 (49.4)	2291 (42.3)	
*Lymphovascular invasion*				<0.001
Absent	125,164 (82.7)	12,206 (75.5)	3671 (67.5)	
Present	7186 (4.7)	2094(12.9)	1132 (20.8)	
Unknown	19,092 (12.6)	1876 (11.6)	637 (11.7)	

Data are expressed as *n* (%) unless otherwise specified

*SLN* sentinel lymph node, *SLNB* sentinel lymph node biopsy, *NOS* not otherwise specified

^a^ Missing data: Breslow (5274), ulceration (5819), immunotherapy (232)

^b^ Not all values add to 100% due to missing data

**Fig. 2:**
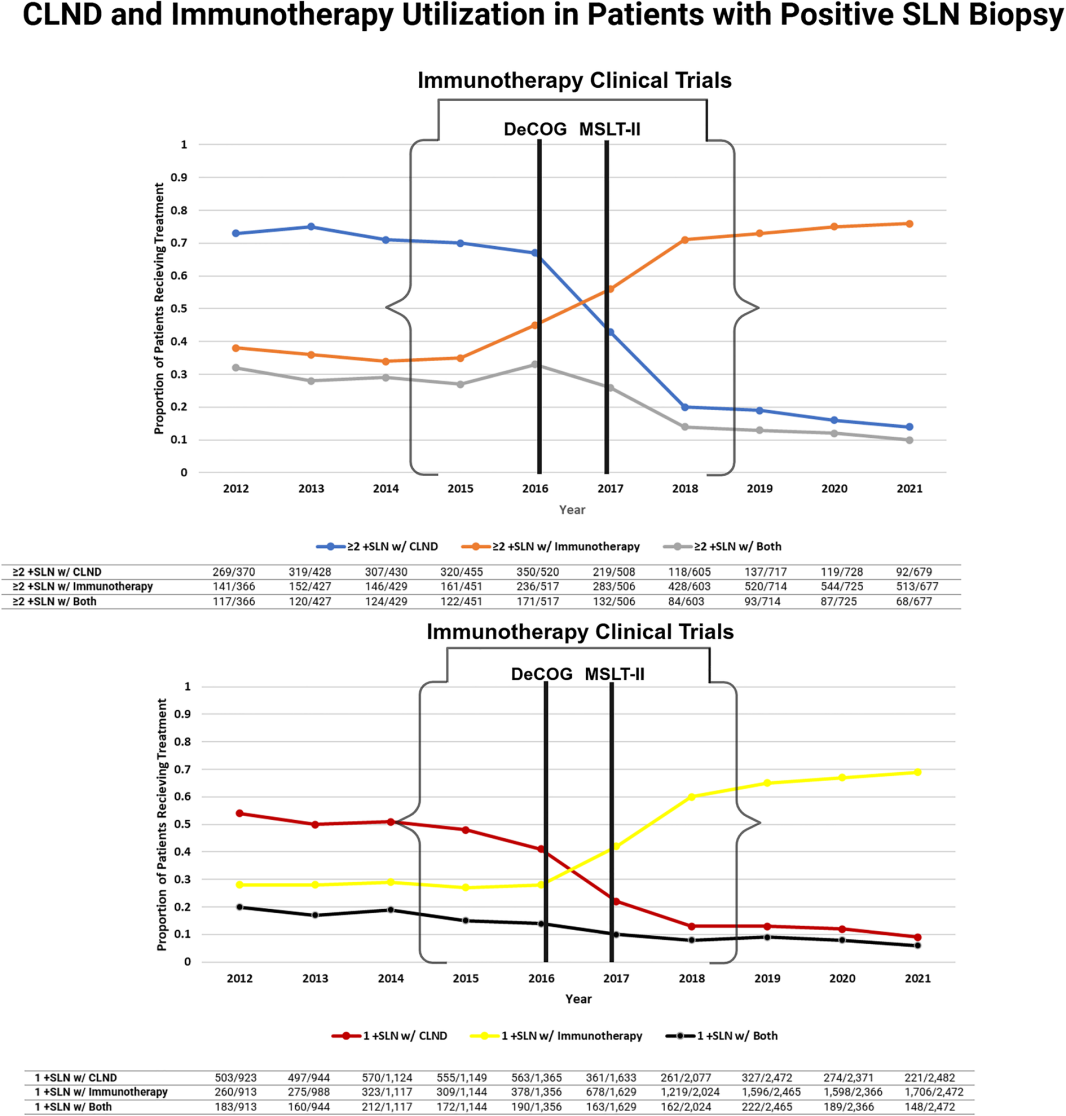
Proportions of patients with 1 or ≥ 2 +SLN and subsequent treatment with CLND and immunotherapy were observed over time. Black vertical bars and brackets denote the year of publication of the DeCOG^6^ and MSLT-II^5^ clinical trials that addressed surgical management of melanoma. Immunotherapy trials addressed medical management of melanoma. Immunotherapy trials include EORTC 18701,^12^ CheckMate 238,^13^ COMBI-AD,^14^ and MK-3475-054/1325-MG/KEYNOTE-054.^15^ The table underneath the graph displays the number of patients for each treatment type in the numerator and the total patients with 1 or ≥ 2 +SLN for each year. The denominators are not equal due to missing data within the immunotherapy variable (*n* = 66 for 1 +SLN and *n* = 25 for ≥ 2 +SLN)

The CLND rates for patients with two or more positive SLNs decreased from 73% in 2012 to 14% in 2021, while the CLND rates for patients with one positive SLN decreased from 54% to 9% during the same time interval (Fig. 2). Immunotherapy rates for patients with two or more positive SLNs increased from 38% in 2012 to 76% in 2021, while the immunotherapy rates for patients with one positive SLN increased from 28% to 69% during the same time interval (Fig. 2).

Between 2018 and 2021, patients with two or more positive SLNs were more likely to undergo CLND if they had head/neck melanoma versus upper extremity melanoma (27.9% vs. 17.3%; OR 2.02, 95% CI 1.39–2.95), ulcerated lesions versus non-ulcerated lesions (18.8% vs. 15.4%; OR 1.42, 95% CI 1.11–1.83), and facilities that were located in the southern United States versus the northeastern United States (17.4% vs. 11.5%; OR 1.77 95% CI 1.06–2.96). Patients were less likely to undergo CLND if they had lower extremity melanoma (i.e., requiring inguinal node dissection) versus upper extremity melanoma (9.6% vs. 17.3%; OR 0.49, 95% CI 0.33–0.72). There was no significant difference between CLND and observation for patients with two or more positive SLNs with regard to sex, age, race, insurance status, Charlson–Deyo score, median income, case volume, facility type, distance traveled, population, Breslow depth, primary tumor site identified as trunk and other location, immunotherapy, or lymphovascular invasion (Table 3).

**Table 3:** Factors associated with undergoing a CLND in patients with two or more positive SLNs (2018–2021; *n* = 2569). Adjustments were made for hierarchical logistic regression modeling with clustering at the hospital level to account for non-independence of samples

	Frequency of undergoing CLND (%)^a^	OR	CI	*p*-Value
*Sex*				
Female	14.1	1.0 (Ref)	–	–
Male	18.9	1.19	0.92–1.54	0.191
*Age, years*				
≥60	15.9	1.0 (Ref)	–	–
<60	18.5	1.24	0.89–1.74	0.198
*Race*				
Non-Hispanic White	17.0	1.0 (Ref)	–	–
Non-White	18.0	1.11	0.71–1.74	0.656
*Insurance status*				
Medicare	16.3	1.0 (Ref)	–	–
No insurance	21.1	1.31	0.63–2.74	0.471
Medicaid	18.3	1.01	0.58–1.76	0.964
Private insurance	17.4	1.04	0.74–1.44	0.832
Other government	17.1	0.91	0.35–2.35	0.838
*Charlson–Deyo score*				
0	17.5	1.0 (Ref)	–	–
1	15.1	0.91	0.63–1.31	0.603
≥2	15.4	0.81	0.49–1.33	0.401
*Median income (quartiles)*				
Lowest	17.8	1.0 (Ref)	–	–
Low	16.9	0.88	0.50–1.54	0.652
High	15.7	1.06	0.60–1.88	0.838
Highest	16.8	0.87	0.49–1.57	0.655
Unknown	18.9	0.93	0.52–1.65	0.797
*Case volume*				
Lowest (<13 cases/year)	16.9	1.0 (Ref)	–	–
Low (13–27 cases/year)	18.1	1.12	0.72–1.75	0.618
Moderate (28–57 cases/year)	18.0	1.03	0.64–1.65	0.915
High (58–111 cases/year)	14.9	0.83	0.48–1.44	0.515
Highest (≥112 cases/year)	17.7	0.90	0.47–1.73	0.753
*Facility type*				
Academic	16.3	1.0 (Ref)	–	–
Non-academic	17.5	1.07	0.74–1.55	0.705
*Distance traveled, miles*				
≤50	16.2	1.0 (Ref)	–	–
>50	18.8	0.98	0.67–1.42	0.901
*Region*				
Northeast	11.5	1.0 (Ref)	–	–
Midwest	17.2	1.56	0.91–2.67	0.105
South	17.4	1.77	1.06–2.96	0.031
West	17.8	1.74	0.98–3.07	0.058
Unknown	21.5	1.99	1.07–3.68	0.028
*Population*				
Metro	17.1	1.0 (Ref)	–	–
Urban	17.0	0.95	0.67–1.37	0.800
Rural	22.5	1.08	0.42–2.76	0.878
*Breslow depth, mm*				
<1.0	15.7	1.0 (Ref)	–	–
1.0–2.0	14.7	1.00	0.58–1.74	0.998
2.01–4.0	15.9	1.05	0.61–1.80	0.867
>4.0	19.9	1.31	0.76–2.25	0.337
*Ulceration status*				
No ulceration	15.4	1.0 (Ref)	–	–
Ulceration	18.8	1.42	1.11–1.83	0.006
*Primary site*				
Upper extremity	17.3	1.0	–	–
Head/neck	27.9	2.02	1.39–2.95	<0.001
Trunk	17.4	0.97	0.69–1.36	0.855
Lower extremity	9.6	0.49	0.33–0.72	<0.001
Other/NOS	20.0	1.78	0.48–6.50	0.387
*Immunotherapy*				
Not given or recommended	17.7	1.0	–	–
Given or recommended	17.0	1.15	0.87–1.51	0.329
*Lymphovascular invasion*				
Absent	16.4	1.0	–	–
Present	19.0	1.18	0.88–1.58	0.257
Unknown	17.8	1.32	0.88–1.98	0.177

*CLND* completion lymph node dissection, *SLNs* sentinel lymph nodes, *OR* odds ratio, *CI* confidence interval, *Ref* reference, *NOS* not otherwise specified

^a^ Missing data: race (8), insurance status (17), median income (513), region (331), population (71), Breslow depth (26), ulceration (44), immunotherapy (10), lymphovascular invasion (269)

**Fig. 3:**
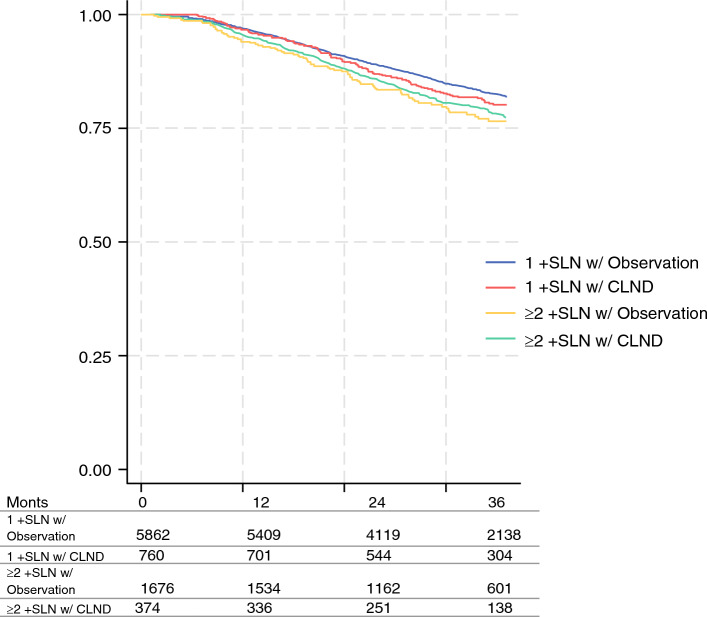
Log rank test: *p* < 0.001. Post-hoc log rank pairwise comparisons of 1 +SLN w/CLND vs. 1 +SLN w/observation: *p* = 0.219. Post-hoc log rank pairwise comparisons of ≥ 2 +SLN w/CLND vs. ≥ 2 +SLN w/observation: *p* = 0.563. The table at the bottom of the figure displays the number alive within each group at each time point

Overall survival of patients with positive SLN disease between 2018 and 2020 was examined (Fig. 3). Pairwise log-rank comparisons between groups demonstrated no significant difference in overall survival between patients with one positive SLN with observation versus one positive SLN with CLND (3-year overall survival 76.5% vs. 74.5%; *p* = 0.219). There was also no significant difference for patients with two or more positive SLNs with observation versus two or more positive SLNs with CLND (3-year overall survival 70.5% vs. 70.8%; *p* = 0.563). A Cox proportional hazards model with clustering at the level of the facility found that the risk of death was not significantly different among patients with two or more positive SLNs who underwent CLND compared with those who had observation (hazard ratio [HR] 1.03, 95% CI 0.77–1.37). Receipt of immunotherapy resulted in a lower risk of death compared with those who were not given, or recommended to receive, immunotherapy (HR 0.72, 95% CI 0.59–0.89). Similarly, the risk of death was decreased for rural location compared with metro location (HR 0.21, 95% CI 0.05–0.95), for those aged <60 years compared with those aged ≥60 years (HR 0.67, 95% CI 0.48–0.92), and centers with high case volume compared with the lowest case volume (HR 0.65, 95% CI 0.47–0.89). The risk of death was increased for those with a Charlson–Deyo score of 2 or higher compared with a score of 0 (HR 2.01, 95% CI 1.48–2.73), the Midwest (HR 1.40, 95% CI 1.00–1.94) and South (HR 1.42, 95% CI 1.03–1.96) compared with the Northeast, and tumor ulceration compared with no ulceration (HR 1.43, 95% CI 1.15–1.78) (Table 4).

**Table 4:** Cox proportional hazard ratios were determined for patients with two or more positive SLNs [2018–2020; *n* = 1928]

Characteristic	Unadjusted 3-year survival (%)^a^	HR	CI	*p*-Value
*Positive SLN response*				
Observation	70.5	1.0 (Ref)	–	–
CLND	70.8	1.03	0.77–1.37	0.867
*Sex*				
Female	73.8	1.0 (Ref)	–	–
Male	68.6	1.13	0.89–1.44	0.321
*Age, years*				
≥60	61.4	1.0 (Ref)	–	–
<60	82.1	0.67	0.48–0.92	0.014
*Race*				
Non-Hispanic White	70.8	1.0 (Ref)	–	–
Non-White	66.9	1.11	0.76–1.64	0.562
*Insurance status*				
Medicare	58.7	1.0 (Ref)	–	–
No insurance	78.7	0.48	0.22–1.04	0.064
Medicaid	72.6	0.94	0.57–1.58	0.825
Private insurance	80.5	0.58	0.43–0.79	<0.001
Other government	82.4	0.63	0.20–1.96	0.426
*Charlson–Deyo score*				
0	73.9	1.0 (Ref)	–	–
1	66.5	1.30	0.98–1.71	0.068
≥2	38.6	2.01	1.48–2.73	<0.001
*Median income*				
Lowest	68.1	1.0 (Ref)	–	–
Low	69.3	0.76	0.50–1.17	0.211
High	65.5	0.93	0.64–1.35	0.702
Highest	73.8	0.87	0.61–1.23	0.425
Unknown	72.6	0.73	0.49–1.08	0.117
*Case volume*				
Lowest (<13 cases/year)	62.7	1.0 (Ref)	–	–
Low (13–27 cases/year)	70.1	0.79	0.56–1.08	0.132
Moderate (28–57 cases/year)	73.8	0.72	0.52–1.01	0.057
High (58–111 cases/year)	73.1	0.65	0.47–0.89	0.008
Highest (≥112 cases/year)	72.6	0.81	0.62–1.07	0.134
*Facility type*				
Academic	70.1	1.0 (Ref)	–	–
Non-academic	71.0	1.11	0.89–1.38	0.361
*Distance traveled, miles*				
≤50	70.1	1.0 (Ref)	–	–
>50	71.5	1.16	0.89–1.50	0.267
*Region*				
Northeast	71.2	1.0 (Ref)	–	–
Midwest	67.2	1.40	1.00–1.94	0.049
South	66.1	1.42	1.03–1.96	0.031
West	74.1	1.02	0.70–1.50	0.901
Unknown	85.8	0.85	0.47–1.55	0.600
*Population*				
Metro	70.4	1.0 (Ref)	–	–
Urban	69.7	0.85	0.63–1.16	0.302
Rural	90.5	0.21	0.05–0.95	0.043
*Breslow depth, mm*				
<1.0	78.7	1.0 (Ref)	–	–
1.0–2.0	80.8	0.77	0.48–1.25	0.295
2.1–4.0	73.2	0.91	0.57–1.45	0.689
>4.0	58.3	1.50	0.94–2.40	0.092
*Ulceration status*				
No ulceration	78.6	1.0 (Ref)	–	–
Ulceration	62.3	1.43	1.15–1.78	0.001
*Primary site*				
Upper extremity	67.9	1.0 (Ref)	–	–
Head/neck	74.2	1.00	0.70–1.43	0.993
Trunk	71.4	1.13	0.84–1.50	0.418
Lower extremity	69.7	1.04	0.76–1.43	0.805
Other/NOS	41.7	2.97	0.97–9.05	0.056
*Immunotherapy*				
Not given or recommended	64.8	1.0 (Ref)	–	–
Given or recommended	72.6	0.72	0.59–0.89	0.002
*Lymphovascular invasion*				
Absent	73.2	1.0 (Ref)	–	–
Present	62.6	1.17	0.92–1.48	0.201
Unknown	69.6	1.02	0.74–1.41	0.902

*SLN* sentinel lymph node, *CLND* completion lymph node dissection, *HR* hazard ratio, *CI* confidence interval, *Ref* reference, *NOS* not otherwise specified

^a^ Missing data: race (4), insurance status (13), median income (370), region (240), population (56), Breslow depth (15), ulceration (33), immunotherapy (8), lymphovascular invasion (215)

Adjustments were made using Cox regression modeling with clustering at the hospital level to adjust for non-independence of samples

## Discussion

Among patients with melanoma, undergoing CLND carries a significant risk of morbidity,^1^ and many patients who underwent CLND were often found not to have additional positive non-sentinel nodes.^2,3^ Thus, trials such as MSLT-II and DeCOG were undertaken to assess whether a CLND improves oncologic outcomes. However, it has been noted that there were relatively few patients with two or more positive SLNs in the MSLT-II and DeCOG trials, thus it is unclear how the trial results were interpreted and how practice patterns may have evolved for patients with two or more positive SLNs. This study found that national practice patterns of CLND in patients with two or more positive SLNs have incorporated large decreases in CLND utilization in concordance with the results from the MSLT-II and DeCOG trials for patients with one positive SLN and patients with two or more positive SLNs. This study also found that there were large increases in immunotherapy utilization for melanoma patients with one positive SLN as well as patients with two or more positive SLNs. Patients with two or more positive SLNs were more likely to undergo CLND if they had tumor ulceration, head/neck location of the primary tumor, and were treated at reporting facilities located in the southern United States, whereas patients with two or more positive SLNs and lower extremity tumor location were less likely to undergo CLND. Among patients with two or more positive SLNs, there was no significant difference in overall survival among those who underwent CLND compared with those who did not.

Reduced CLND use has been a trend that predated the release of MSLT-II or DeCOG data.^25–28^ In this study, the rate of CLND use for patients with melanoma and one positive SLN decreased significantly, and an even greater absolute decrease was observed in the rate of CLND for patients with two or more positive SLNs. This change in treatment patterns coincides with an increase in immunotherapy within the same groups of patients, as an effective adjuvant therapy likely gives even greater comfort in observing patients with multiple positive SLNs rather than performing a CLND. The MSLT-II and DeCOG trials accelerated avoidance of CLND and this happened in a relatively short period of time, defying the often-quoted 17 years needed to get trial findings into practice.^29^

In 2018, the NCDB introduced a new variable describing SLN positivity, which has made analysis of patients with increased sentinel node disease more accurate. Other similar NCDB analyses were limited prior to the release of this variable.^25,26^ In the current study, we found that between 2018 and 2021, patients with two or more positive SLNs were more likely to undergo CLND if the tumor was located on the head/neck and if the lesion was ulcerated. Similar results have been reported regarding increased CLND utilization in patients with melanoma with tumors located on the head/neck when examining United States^30^ and international populations.^31^ One hypothesis is that these patients may be managed by otolaryngologists who might have different practice patterns and be more inclined to perform CLND. Patients were also more likely to undergo CLND if the reporting facility was located in the southern United States. Regional variation in SLNB^32^ as well as CLND^20^ across the United States has been described, however there is no clear pattern of regional differences in the rates of these procedures. Rates of CLND and adjuvant immunotherapy also vary between the United States and other high-volume melanoma centers around the world.^31^ Conversely, patients were less likely to undergo CLND if they had lower extremity melanoma. Inguinal dissection is required to treat lower extremity melanoma, a procedure with greater complexity and risk of lymphedema compared with other CLND procedures (e.g., axillary),^33^ thus surgeons and patients may have shied away from inguinal CLND but remain more comfortable with CLND of other nodal basins. Rates of CLND were lower in the northeast compared with other regions. The odds of CLND in the south were significantly lower than in the northeast. It has been shown that residents in the northeast have the greatest access to immunotherapy clinical trials in melanoma^34^ and this may have also impacted the rates of CLND.

The decline in rates of CLND use began around 2015 with the increasing publication of melanoma immunotherapy clinical trials, and the decline became more pronounced in 2016–2017 following publication of the DeCOG and MSLT-II trials. Although both trials were primarily composed of patients with one positive sentinel node, with relatively few patients with two or more positive SLNs, our findings show that clinicians in the United States have also applied these results to patients with more than one positive sentinel node. Given the small numbers of patients with two or more positive SLNs in both trials, the results could not be separately assessed for patients with two or more positive SLNs.

The results of the MSLT-II and DeCOG trials have validated nodal observation as a suitable alternative to CLND for patients who have one positive SLN, with similar melanoma-specific survival comparing observation against CLND.^5,6^ Several other studies have investigated the NCDB and confirmed adoption of these practice patterns for melanoma patients in the United States with positive SLNs; however, these studies did not separately analyze patients with two or more positive SLNs.^20,27^ Similar to our findings in this study, survival has not been found to be significantly different for patients who underwent CLND compared with those who were observed.^20,35^ Melanoma-specific survival, SLN-basin only recurrences, and all-site recurrence may also be similar when comparing patients with high-risk melanoma who undergo surveillance compared with CLND.^36^ Although further investigation of disease recurrence and patient survival is required, these results are reassuring in that reduced CLND utilization in patients with melanoma with two or more positive SLNs was not associated with lower overall survival. It is important to reiterate that this change was likely influenced by concomitant uptake of adjuvant immunotherapy in this population.

The strengths of our study include those inherent to the NCDB, such as standardized data collection and abstraction methods, which allows for accurate comparisons across numerous diverse health systems across the country. Considerable time has elapsed since the dissemination of findings from the MSLT-II and DeCOG clinical trials, allowing assessment of subsequent practice patterns and patient survival. In addition, our study was conducted after introduction of a variable into the NCDB that allows for quantification of positive SLNs.

Our study also has several limitations. First, the NCDB is unable to determine cancer recurrence and specific survival measures such as relapse-free survival, distant metastasis-free survival, and melanoma-specific survival. More granular measurements of survival (e.g., melanoma-specific survival) are particularly relevant to the treatment of melanoma as MSLT-II found that disease-free survival was improved in CLND compared with observation.^5^ The DeCOG trial,^6^ as well as the study by Broman et al.,^36^ found that there were no differences in recurrence-free survival comparing CLND with observation. Immunotherapy trials have shown decreased recurrence-free survival in patients who are treated with adjuvant therapy.^13–15^ We observed sharp declines in CLND with increases in immunotherapy, however both therapies may improve disease-free survival. Second, the NCDB only includes information from CoC institutions and these may not represent melanoma treatment patterns of non-CoC-accredited facilities. Given the requirements to be a CoC-accredited center, non-CoC-accredited centers may have different practice patterns and adherence to established guidelines. Third, there is no detailed information in the NCDB on the size of the nodal deposit(s) and this may be an important factor in treatment decisions and prognosis. Finally, the NCDB had yet to release data for 2022–2024 at the time of this study and we were unable to assess the most up-to-date patterns of CLND and immunotherapy at the time of this publication.

In this study of patients with melanoma, the rates of CLND decreased for patients with two or more positive SLNs on biopsy; this change in practice patterns occurred following publication of two pivotal clinical trials. Clinicians who provide care for patients with melanoma may be informed by these national trends in management, and those who routinely offer CLND may reconsider their recommendations given these findings of reduced utilization with comparable overall survival with observation. Likewise, clinicians may offer immunotherapy for appropriately selected patients with melanoma, which has had a substantial increase in utilization for patients with melanoma. The use of CLND continues to be evolving, given that even neoadjuvant immunotherapy for positive clinical nodal disease may resolve all disease. The results of this study showing that overall survival between observation and CLND for patients with melanoma with two or more positive SLNs was not significantly different provides reassurance that the decision to observe the nodal basin, with adjuvant therapy, may lead to acceptable overall survival.

## Conclusion

Although patients with two or more positive SLNs were infrequent in the MSLT-II and DeCOG trials, the results of these studies have been extended to this patient population in the United States. Use of CLND in melanoma patients with two or more positive SLNs has declined and has been met with comparable outcomes to CLND.

## Supplementary Information

Below is the link to the electronic supplementary material.

Supplementary file 1 (DOCX 14 kb)
