# Supplementary material for: National Evaluation of the Management of Melanoma Patients with Multiple Positive Sentinel Lymph Nodes
Source: Ann Surg Oncol. 2025 Nov 1;33(1):397–410. doi: 10.1245/s10434-025-18466-4 (PMC12689804; doi:10.1245/s10434-025-18466-4)
Supplement: Supplementary file 1 — (DOCX 14 kb) [file 10434_2025_18466_MOESM1_ESM.docx]

**SUPPLEMENTARY TABLE 1** Rates of treatment per year for stage III melanoma

*Stage IIIA*

| Year | 2012 | 2013 | 2014 | 2015 | 2016 | 2017 | 2018 | 2019 | 2020 | 2021 |
| --- | --- | --- | --- | --- | --- | --- | --- | --- | --- | --- |
| CLND | 77/112(69%) | 98/131(75%) | 99/140(71%) | 106/154(69%) | 112/169(66%) | 70/190(37%) | 20/140(14%) | 18/130(14%) | 23/134(17%) | 14/131(11%) |
| Immunotherapy | 41/112(37%) | 50/131(38%) | 43/140(31%) | 52/154(34%) | 79/169(47%) | 103/190(54%) | 97/140(69%) | 92/130(71%) | 96/134(72%) | 96/131(73%) |
| Both | 34/112(30%) | 41/131(31%) | 41/140(29%) | 42/154(27%) | 57/169(34%) | 38/190(20%) | 13/140(9%) | 9/130(7%) | 17/134(13%) | 9/131(7%) |

*Stage IIIB*

| Year | 2012 | 2013 | 2014 | 2015 | 2016 | 2017 | 2018 | 2019 | 2020 | 2021 |
| --- | --- | --- | --- | --- | --- | --- | --- | --- | --- | --- |
| CLND | 97/127(76%) | 101/142(71%) | 98/134(73%) | 107/155(69%) | 125/192(65%) | 71/191(37%) | 11/127(9%) | 24/143(17%) | 16/147(11%) | 11/125(9%) |
| Immunotherapy | 55/127(43%) | 51/142(36%) | 54/134(40%) | 53/155(34%) | 81/192(42%) | 105/191(55%) | 98/127(71%) | 102/143(71%) | 115/147(78%) | 101/125(81%) |
| Both | 50/127(39%) | 38/142(27%) | 42/134(31%) | 39/155(25%) | 60/192(31%) | 44/191(23%) | 9/191(7%) | 16/143(11%) | 12/147(8%) | 8/125(6%) |

*Stage IIIC*

| Year | 2012 | 2013 | 2014 | 2015 | 2016 | 2017 | 2018 | 2019 | 2020 | 2021 |
| --- | --- | --- | --- | --- | --- | --- | --- | --- | --- | --- |
| CLND | 76/103(74%) | 101/125(81%) | 91/118(77%) | 95/123(77%) | 112/153(73%) | 77/123(63%) | 69/286(24%) | 81/385(21%) | 57/381(15%) | 54/357(15%) |
| Immunotherapy | 38/103(37%) | 41/125(33%) | 41/118(35%) | 50/123(41%) | 73/153(48%) | 73/123(59%) | 209/286(73%) | 285/385(74%) | 290/381(76%) | 268/357(75%) |
| Both | 29/103(28%) | 33/125(26%) | 37/118(31%) | 41/123(33%) | 57/153(37%) | 50/123(41%) | 51/286(18%) | 62/385(16%) | 46/381(12%) | 43/357(12%) |

* Stage IIID was only implemented after the AJCC 8th edition in 2018 and was not included in this analysis
